# MENOS4 trial: a multicentre randomised controlled trial (RCT) of a breast care nurse delivered cognitive behavioural therapy (CBT) intervention to reduce the impact of hot flushes in women with breast cancer: Study Protocol

**DOI:** 10.1186/s12905-018-0550-z

**Published:** 2018-05-08

**Authors:** Deborah Fenlon, Jacqueline Nuttall, Carl May, James Raftery, Jo Fields, Emma Kirkpatrick, Julia Abab, Mary Ellis, Taylor Rose, Priya Khambhaita, Angeliki Galanopoulou, Tom Maishman, Jo Haviland, Gareth Griffiths, Lesley Turner, Myra Hunter

**Affiliations:** 1 College of Human and Health Sciences, Swansea University, Swansea, UK; 2 Faculty of Health Sciences, University of Southampton, Southampton, UK; 3 Southampton Clinical Trials Unit, University of Southampton, Southampton, UK; 4 Primary Care and Population Sciences, University of Southampton, Southampton, UK; 5 Poole Hospital NHS Trust, Poole, UK; 6 Independent Cancer Patients’ Voices, London, UK; 7 King’s College, London, London, UK; 8 Royal Marsden Clinical Trials Unit, Sutton, UK; 9 Pensions Policy Institute, King’s College, WC2B 6LE, London, UK

**Keywords:** Breast cancer, Menopause, Hot flushes, Night sweats, CBT, Training, Nurses, Normalisation process theory

## Abstract

**Background:**

Women who have been treated for breast cancer may identify vasomotor symptoms, such as hot flushes and night sweats (HFNS), as a serious problem. HFNS are unpleasant to experience and can have a significant impact on daily life, potentially leading to reduced adherence to life saving adjuvant hormonal therapy. It is known that Cognitive Behavioural Therapy (CBT) is effective for the alleviation of hot flushes in both well women and women who have had breast cancer. Most women with breast cancer will see a breast care nurse and there is evidence that nurses can be trained to deliver psychological treatments to a satisfactory level, whilst also maintaining treatment fidelity. The research team will assess whether breast care nurses can effectively deliver a CBT intervention to alleviate hot flushes in women with breast cancer.

**Methods:**

This study is a multi-centre phase III individually randomised controlled trial of group CBT versus usual care to reduce the impact of hot flushes in women with breast cancer. 120–160 women with primary breast cancer experiencing seven or more problematic HFNS a week will be randomised to receive either treatment as usual (TAU) or participation in the group CBT intervention plus TAU (CBT Group).

A process evaluation using May’s Normalisation Process Theory will be conducted, as well as practical and organisational issues relating to the implementation of the intervention. Fidelity of implementation of the intervention will be conducted by expert assessment. The cost effectiveness of the intervention will also be assessed.

**Discussion:**

There is a need for studies that enable effective interventions to be implemented in practice. There is good evidence that CBT is helpful for women with breast cancer who experience HFNS, yet it is not widely available. It is not yet known whether the intervention can be effectively delivered by breast care nurses or implemented in practice. This study will provide information on both whether the intervention can effectively help women with hot flushes and whether and how it can be translated into routine clinical practice.

**Trial registration:**

ISRCTN 12824632. Registered 25–01-2017.

**Electronic supplementary material:**

The online version of this article (10.1186/s12905-018-0550-z) contains supplementary material, which is available to authorized users.

## Background

Hot flushes, also known as hot flashes, and night sweats (HFNS) are experienced by up to 70% of women after treatment for breast cancer [29]. HFNS are troublesome for many women, having a significant impact on daily life and sleep quality, with important social consequences affecting employment, personal relationships and quality of life [17]. With natural menopause, HFNS gradually decrease in number and intensity over the post-menopausal years. With breast cancer, HFNS can be more extreme and persistent: experienced by 34% of women more than five years after diagnosis and by 50% of women more than five years from menopause [16]. This is due in part to treatments for breast cancer, which reduce or interfere with the action of oestrogen in the body. Chemotherapy may precipitate an early menopause [30] and tamoxifen and other hormonal treatments cause or exacerbate HFNS [32]. Adjuvant hormone therapies may be used sequentially for a minimum of five years and now up to ten years post diagnosis. The majority of women do not complete the recommended five years of adjuvant hormone therapy, which may be partly due to adverse side effects, such as HFNS, resulting in a 30% increased breast cancer mortality [25, 28].

The most effective treatment for HFNS is hormone replacement therapy, which is contraindicated in ER+ breast cancer [31]. While there are other medications available, such as selective serotonin re-uptake inhibitors (SSRIs) and clonidine, they have unpleasant side effects, and non-medical alternatives tend to lack efficacy [7]. Furthermore, many women prefer not to take medication after cancer, but instead favour self-management of menopausal HFNS [34]. Surveys carried out by the National Cancer Research Institute (NCRI) breast symptoms working party showed that there are no consistent standard care pathways for people with HFNS and that very few women are offered anything in the way of care or management of this problem [15].

There is evidence that a structured cognitive behavioural therapy (CBT), focusing on key elements of the experience of hot flushes and night sweats, delivered in group format, is effective for the alleviation of HFNS in both women with and without breast cancer [1, 12, 26]. In line with the Medical Research Council’s guidance on developing and evaluating complex interventions [11] the theoretical basis and the role of moderators and mediators of the outcomes of CBT have been examined in previous studies. Hunter and Mann [22] developed a theoretical model of HFNS that draws upon symptom perception, self-regulation and cognitive behavioural theories to explain women’s cognitive appraisal and behavioural reactions to symptoms. The model was tested using structural equation modelling [21] and an examination of mediators in MENOS trials [10]. The results clearly suggest that problem rating of HFNS (i.e. the impact of HFNS on daily life) is mediated mainly by beliefs about HFNS, and that changes in beliefs, as well as improvements in mood and sleep, predict positive outcomes with CBT.

There may be a need to develop a variety of ways to deliver the CBT intervention, but group sessions, led by a health professional such as the breast care nurse (BCN), provide a cost effective solution, and were positively viewed in the previous MENOS trial [2]. There are also benefits with group CBT, such as improvements in mood and quality of life, which have not been demonstrated with self-help CBT [1]. Although CBT is known to be effective, it is rarely offered within the NHS for women with breast cancer. It is also not known whether this intervention can be effectively delivered by BCNs in the NHS context. Most women with breast cancer will see a BCN and there is evidence to suggest that it is possible to train BCNs to deliver psychological interventions to a satisfactory level and fidelity [24]. This study will therefore test whether breast care nurses can be trained to deliver CBT in an NHS context to effectively manage HFNS in women who have had breast cancer.

A further consideration is that practical barriers can prevent effective interventions from being delivered in practice, so we will conduct a process evaluation, drawing on May’s Normalisation Process Theory (NPT), [33], to explore potential barriers to implementation. The theory focuses on the dynamic processes that lead to innovations being implemented and integrated into work on an every-day basis. It is therefore a helpful way to assess what happens when multifaceted interventions are introduced into practice. This includes what people’s actions are, collectively and individually, and how and why the desired outcomes are met (or not). The processes that take place when people implement change are described by NPT as coherence, cognitive participation, collective action and reflexive monitoring. The intervention in this trial is an alternative care pathway that includes CBT. The purpose of the process evaluation is to understand the dynamics of the care pathway and identify factors that are important for embedding this intervention into practice. This evaluation will focus on identifying and explaining the extent to which the planned CBT is implemented into practice.

HFNS have been identified as a major physical symptom by the breast cancer research gap analysis [13], requiring research that identifies appropriate interventions to enable women to manage this problem. The research gap analysis also highlights an inadequate translation of research findings into clinical practice and specifically the need to consider how interventions such as CBT can be better integrated to widen access.

## Methods/Design

The study design is a randomised controlled trial (RCT), with a formal process evaluation. The RCT will be a multi-centre phase III individually randomised controlled trial of a BCN-delivered group CBT intervention versus treatment as usual (TAU).

### Study aims and objectives

The primary study aim is to evaluate the effectiveness of group CBT delivered by breast care nurses on reducing the impact of HFNS in women with breast cancer 26 weeks after randomisation.

Secondary aims include outcome and process issues. These are to explore and evaluate:

1. The extent to which there is a reduction of the impact of HFNS nine weeks after randomisation in women with breast cancer
2. The extent to which there is a reduction of the frequency of hot flushes and night sweats nine and 26 weeks after randomisation in women with breast cancer
3. The level of fidelity of the CBT when delivered by breast care nurses
4. The effect of group CBT on quality of life and other symptoms, e.g. sleep, anxiety
5. The effect on women’s hot flush beliefs and behaviours
6. An estimate of the cost-effectiveness
7. The extent to which the planned CBT intervention was implemented into practice, specifically:
  Exploring how and in what ways the therapy was initially received, how individually and collectively people practically conceptualised and made sense of it (coherence)
  Assessing the degree of ownership of and participation in the new practice by key individuals (surgeons, managers, BCNs and patients) and teams (cognitive participation)
  Identifying the individual and teamwork carried out to sanction the new practice (collective action)
  Exploring the perceived impact of the new practice on staff work and on patient outcomes (reflexive monitoring)

### Study setting

Participants will be recruited from six NHS Hospital Trusts in England and Wales. These centres will be selected from those who express an interest through the NIHR Clinical Research Network (CRN) and will have:

1. Availability of at least two BCNs willing to be trained
2. Available room to deliver the CBT sessions
3. Written agreement to participate from the manager

### Participants

120–160 women with primary breast cancer experiencing seven or more problematic HFNS a week will be recruited and randomised to the intervention or usual care.

Inclusion criteria are:

1. Women with primary breast cancer or ductal carcinoma in situ (DCIS)
2. Women who have completed all primary treatment: surgery and/or radiotherapy and/or chemotherapy (may still be receiving adjuvant endocrine therapy or Herceptin)
3. Aged 16 years or older
4. Experiencing seven or more HFNS/week with an overall rating of 4/10 or above on the Hot Flush Problem Rating Scale
5. Ability to attend group sessions
6. Signed informed consent

The exclusion criteria are:

1. Benign breast disease
2. Metastatic disease (our patient representatives advised us that the group dynamics could be dominated by issues of recurrence and disease progression instead of focusing on HFNS if people with metastatic disease were included)
3. Current use of other mind-body therapies to help with HFNS, e.g. acupuncture, hypnosis and mindfulness.

There will be no exclusion criterion relating to time since diagnosis as long as participants have problematic HFNS. Women who are taking medication or herbal remedies for HFNS will be asked to continue with these throughout the study.

### Study processes

Due to the pragmatic nature of the study, and to emulate the real world situation of this intervention, potential participants will be identified and recruited as flexibly as possible. Therefore, routes of identifying eligible women will include identification from breast cancer follow up clinics, phone clinics, leaflets and posters in clinics and health and wellbeing events, by the research nurses, who will check eligibility and take consent following GCP guidelines.

### Randomisation

Randomisation will be in cohort groups and stratified by site. A computer-generated randomisation sequence will be created by a statistician at the Clinical Trials Unit, allocating participants in a one-to-one ratio, stratified by site with fixed block size. This process will be repeated for each cohort group so that allocation does not affect the allocation sequence of subsequent cohorts. Following receipt of consent and completed baseline case report forms (CRFs) from 12 to 16 eligible participants at a single site participants will be randomised to either Group CBT or TAU. The research nurse will be sent the allocation results for all the women at one time point and they will inform each participant of their group allocation (CBT or TAU). Each site will aim to run two sequential groups of the intervention of 6–8 women per group (NB. A possible group of five (ten women recruited) was also possible via TMG approval).

### Intervention

Women in the intervention arm will attend weekly group CBT sessions, lasting 90 min each, for six weeks. Sessions will be delivered by BCNs who have been trained by a clinical psychologist. The sessions will follow a structured manual [20], which includes psycho-education and the cognitive behavioural model; stress management; paced breathing; cognitive and behavioural strategies to improve wellbeing and for managing hot flushes; night sweats and sleep; and maintaining changes.

### Training

The BCNs involved in delivering the intervention will be selected by sites and will be trained by a clinical psychologist to deliver the intervention. The nurses will be trained as close as practically possible to delivery of the intervention. Training will take place over two days, with 6 h of training per day and an overnight stay in between. Knowledge and skills will be assessed throughout the training using a variety of methods, including questionnaires and role-plays. A telephone based top-up session will be conducted immediately prior to the first group at each site (within three weeks) in order to refresh the learning.

BCNs will use a manual [20] which contains detailed session content, presentation slides and handouts, and notes for facilitators. This will be sent to BCNs in advance of the training days with tasks to complete in preparation, including a sleep diary and relaxation CD. The training will provide the background theoretical knowledge and practical skills to facilitate group CBT for menopausal symptoms by examining how thinking and behaviour can have a significant impact on women’s experience of HFNS following breast cancer treatment and helping women to develop strategies to manage them. These include understanding negative emotions and HFNS, managing unhelpful thoughts and behaviour, improving sleep and using paced breathing to manage HFNS.

### Supervision

BCNs will receive ongoing supervision of their delivery of group CBT. They will be asked to write down their reflections and any questions/problems after each session they deliver and email it to the clinical psychologist who trained them for supervision. Feedback on these reflections will be made by email, telephone or Skype. They can also refer back to the manual. Data will be collected on the number and length of supervisory sessions.

### Adherence

Adherence to group CBT will be measured by the number of sessions attended and the number of times that a participant reports practising relaxation and paced breathing each week. If participants do not attend a session, the BCN will contact the participant by telephone to ascertain the problem of attendance, and will discuss the appropriate solution with the participant e.g. a telephone session. Alternatively, the session is recorded as did not attend. Telephone sessions will be kept to a minimum, and only arranged if exceptional circumstances do not allow the patient to attend the face-to-face session.

### Fidelity

All group sessions will be audio recorded (with consent), and 17% will be randomly selected (with a computer-generated random number sequence), ensuring two sessions per site are selected. An independent psychologist (i.e. who has not been involved in BCN training), experienced in CBT for HFNS, will rate them for adherence to the treatment manual.

### Treatment as usual arm

It is expected that TAU will be different at each site as there is no current standard of care. Since randomisation will be stratified by site this does not pose a problem. In some centres, women will be given ad hoc advice about HFNS, normally only if they raise the issue. Data collected from a UK survey suggests that only 29% women were asked if they were experiencing HFNS, only 2% were referred to a menopause clinic and very few offered any kind of relaxation or behavioural intervention [15].

In addition to standard NHS care, participants randomised to the TAU arm will be offered a version of self-help CBT following the 26-week assessment. This involves giving women a booklet and CD that includes the same information as group CBT sessions, as well as a one-to-one face-to-face meeting with a trained BCN to discuss the key elements of the booklet. This will be followed up by two telephone calls to discuss progress, encourage use of the booklet and homework and to address any problems. Offering self-help CBT will be used as a strategy to increase adherence to the study.

### Outcome measures

Outcome measures will be completed at baseline, week nine and week 26. Baseline demographic and clinical information, including use of current therapies, will be collected and input on the database. A team from the Clinical Informatics Research Unit at the University of Southampton will develop the trial database. The infrastructure will be provided by ALEA.

#### Hot flushes and night sweats assessment

The primary study outcome will be measured using the Hot Flushes and Night Sweats (HFNS) Problem Rating Scale [18]. This measures the extent to which hot flushes and night sweats are problematic, distressing and interfere with daily life. Three items are rated on a 10-point scale – higher scores are indicative of greater bother/impact on daily life. A change of 2 points on this scale is considered clinically relevant [1, 26]. This scale also assesses HFNS frequency asking women to estimate how many HFNS they have had in the past week. A three day diary will be collected at baseline to validate the accuracy of the estimate.

The Short Form Hot Flush Beliefs and Behaviours Scale (HFBBS) is a 16-item scale that includes items about beliefs and behaviours about hot flushes [19]. Subscales include: (i) beliefs about HF in social context (e.g. everyone is looking at me), (ii) beliefs about coping/control of hot flushes (e.g. when I have a HF I think they will never end), and (iii) beliefs about night sweats and sleep (e.g. if I have NS I’ll never get back to sleep). HFNS Behaviours include (i) positive coping behaviour, e.g. accepting HFNS, using breathing and calming responses; (ii) avoidance behaviour.

The Hot Flash Related Daily Interference Scale (HFRDIS) [9] measures the impact of hot flushes on a variety of domains including work, social, and leisure activities on a scale from 0 to 10.

#### Quality of life

QoL will be assessed using the EQ-5D-5 L and the FACT-B [4]. FACT -B is a widely used and well-validated 37-item questionnaire designed for use in breast cancer. Five sub-scales assess physical, social, emotional and functional well-being, as well as concerns specific to women with breast cancer. The endocrine subscale (ES) [14] includes 19 items related to hormone treatment.

#### Anxiety and depression

The Generalised Anxiety Disorder Questionnaire (GAD-7) is a self-administered patient questionnaire consisting of seven items (e.g. feeling nervous, restlessness) used as a severity measure for generalised anxiety disorder [35].

The Patient Health Questionnaire-9 (PHQ-9) is a measure of depressive mood. It is used to examine the severity of depression and response to treatment. It is self-administered and patients are asked how often they have been bothered by nine problems (e.g. trouble concentrating and poor appetite) over the previous two weeks [23].

#### Sleep

The Pittsburgh Sleep Quality Index (PSQI) is a self-rated questionnaire that assesses sleep quality and disturbances from 19 individual items, including sleep quality, sleep latency, sleep duration, habitual sleep efficiency, sleep disturbance, sleep medication use and daytime dysfunction (Bussye et al. [6]). These are analysed in seven component scores with the sum of scores yielding one total score. This has been validated for use in women with breast cancer [8].

#### Health economics assessment

The economic analysis will estimate the costs of providing the CBT intervention, and the cost consequences of the intervention for NHS services and for costs borne by patients. However, an economic evaluation will be conducted only if the intervention proves to be effective, as defined by a two-point improvement in the HFNS. The cost of the intervention will be reported regardless of its effectiveness. If effective, cost effectiveness will be expressed in cost per unit change in HFNS and per QALY, based on the incremental differences between arms. The identification and collection of costs will be undertaken using the following methods:

1) NHS

Data on the use of medication, primary care visits, and out-patient visits will be collected using a resource use questionnaire. The cost of the intervention will be based on nurse logs to record staff training cost, and time to deliver the intervention. This information will be used for sensitivity analysis from a societal perspective. We will use an adapted form of the client service receipt inventory (CSRI), Beecham & Knapp, [3].

2) Women

We will estimate out-of-pocket spending such as herbal remedies, acupuncture or alternative therapies and time off work due to hot flushes. Collection of such information from each participant will be through a resource use questionnaire at 9 and 26 weeks. QoL will be measured by EQ-5D-5 L and will be collected at baseline, 3, 6, 9 and 26 weeks.

### Process evaluation

An evaluation questionnaire will be administered to those participants in the Group CBT intervention arm at the end of the six-week intervention. Interviews will be conducted with patients and key stakeholders from each of the study centres at the completion of the intervention. Semi-structured interview schedules will be developed, guided by consideration of the four areas identified through NPT [33], which includes an exploration of barriers to implementation and how they were tackled. Key stakeholders will include all the participating BCNs and 1 key manager and 1 key member of clinical staff at each site identified by the BCNs. Interviews will be conducted with BCNs prior to, and after delivery of the intervention. All other stakeholders will be interviewed after the intervention. Interviews will be either face-to-face or by telephone.

### Analysis

#### Sample size calculation

A difference of two points or more in the HFNS Problem Rating Scale is regarded as clinically relevant. In order to detect a two-point difference (standard deviation 2.4; standardised effect size 0.8, [26] in mean HFNS problem rating for the comparison of CBT to TAU, 90% power would require 64 participants in total (32 per randomised arm), assuming 2-sided significance level of 0.05. Allowing for an inflation factor of 1.49 (intraclass correlation of 0.07 with 8 participants per group, (Wampold and Brown [36]) to adjust for expected clustering of outcomes within groups, gives a minimum sample size of 96, which increases to 120 allowing for 20% loss to follow-up. A sample size of 120 will also allow each site to run two groups to ensure that a comprehensive process evaluation can be conducted. If each site recruits the minimum number of six people per group, then 120 participants in total will be achieved. If they recruit the maximum of eight per group (allowing up to 160 participants in total) this will provide greater power for the analyses of secondary outcomes.

#### Primary and secondary analyses

Scales from the validated questionnaire measures will be calculated according to published scoring algorithms. The difference in the HFNS Problem Rating Scale (primary outcome) between the two randomised groups will be tested using a linear mixed model, utilising fixed and random effects. The regression model will compare the HFNS problem rating subscale between intervention groups at follow-up, adjusting for baseline HFNS problem rating score and stratification factor (site). Greater precision of estimates is expected within therapy groups (clustering effect) so models will also be adjusted for the group. Secondary outcomes at post-treatment will be analysed in a similar way. Follow-up data at subsequent time intervals will also be explored through linear mixed models utilising repeated measures analyses, allowing simultaneous modelling of the three outcome time points. Analyses will be based on a modified intention-to-treat sample (i.e. excluding participants who contribute fewer than two items on the primary outcome measure). Per protocol analysis for those compliant will be performed as a sensitivity analysis.

#### Health economics analysis

All relevant resource items identified will be costed using published national cost data (British National Formulary and Personal Social Services Research Unit, and NHS reference cost). Accumulated costs and quality adjusted life years (QALYs) per patient will be estimated by means of area under the curve. Where appropriate we will estimate incremental cost-effectiveness ratios. We will estimate mean values and 95% percentiles using non-parametric bootstrapping. We will produce cost-effectiveness acceptability curves to illustrate the uncertainty of such estimates. Major assumptions made in the costing and QALYs will be tested by means of sensitivity analyses.

#### Qualitative analysis of process evaluation

Interview recordings will be transcribed and all identifying data will be anonymised. The data will be analysed using thematic analysis [5] and the principles of open coding, constant comparison, negative case analysis, and memo writing [27]. In addition, some a-priori codes derived from the literature review will be used. The NVivo 10 software program will be used to facilitate data storage, categorisation and retrieval. Members of the research team will code the interviews, hold coding meetings, and revise the coding strategy. After coding, themes will be proposed and tested in the data. Analysis meetings with the research team will involve refining the themes.

Fidelity of delivery of the therapy will be assessed from randomly selected audio recordings. An experienced, independent clinical psychologist will indicate on coding sheets the extent to which the group leader covered each topic, using the Quality Assurance for Group CBT intervention Independent Sessional Assessment tool used in MENOS study 1 [26]. Coding sheets include specific components of the intervention (e.g. reviewing homework, providing information about the role of stress, demonstrating paced breathing in the session, group discussion of behaviours relating to HFNS) developed for the trial.

## Discussion

There is a need for studies that enable effective interventions to be implemented in practice. There is good evidence that CBT is helpful for women with breast cancer [26], yet it is not widely available. It is not yet known whether the intervention can be effectively delivered by BCNs. This study will provide information on both whether the intervention can effectively help women with HFNS and whether and how it can be translated into routine clinical practice.

**Fig. 1:**
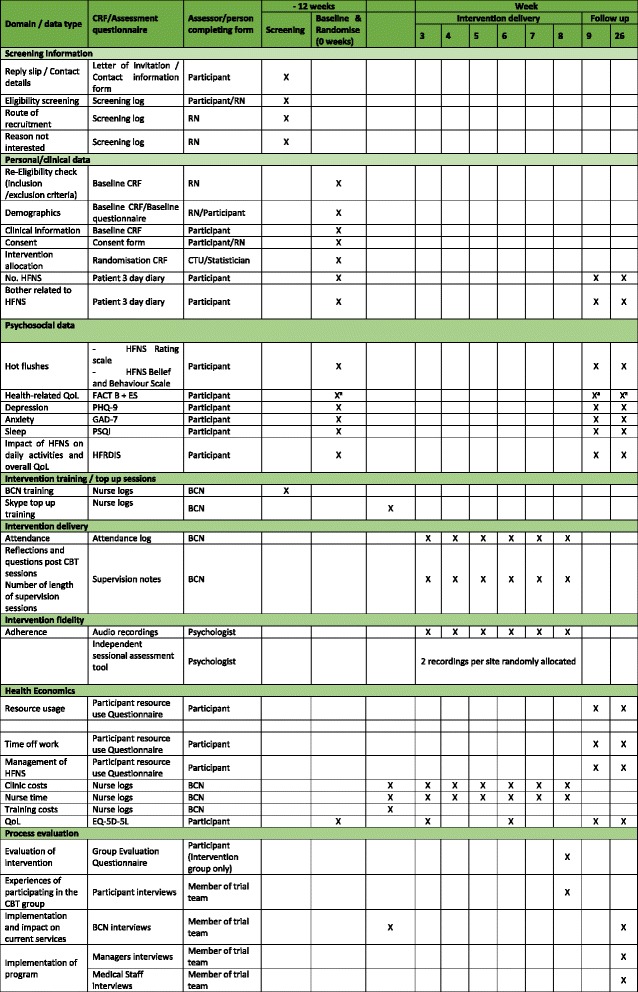
Schedule of observations and procedures

**Fig. 2:**
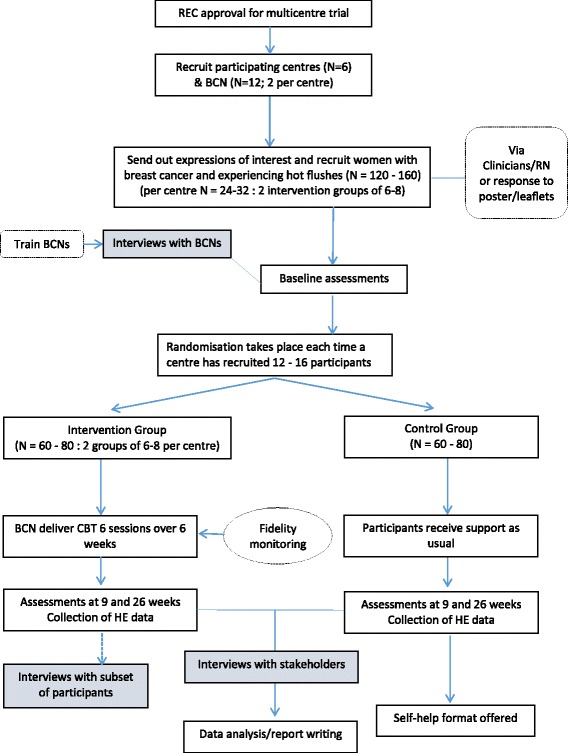
Study Schema

Despite the fact that CBT has been demonstrated to be effective in relieving the bother of HFNS in women who have had breast cancer, it is not widely available and is rarely offered to women who are suffering these symptoms. One reason for this could be the relatively small number of therapists available to offer this kind of intervention. Most women with breast cancer have access to a BCN and nurses can be effectively trained to deliver psychological interventions. If it can be shown that this intervention can be successfully delivered by BCNs this could make the intervention available more widely. However, there are often other barriers to the implementation of effective therapies, so this study will provide a qualitative evaluation of challenges and barriers and how participating centres overcame these to implement this service into their practice (Figs. 1, 2 and Additional file 1).

### End of the trial

The end of trial is defined as when the last patient has had their last data collected.

### Trial status

This clinical trial was registered in January 2017 (ISRCTN (12824632)). Recruitment opened in January 2017 and is expected to be completed by March 2018. The current protocol is version 3, dated 21-April-2017. Results will be published at the end of the trial in a peer reviewed journal (authored by the members of the TMG), presented at international conferences, end of trial summaries will appear on the relevant databases and results fed back to recruiting sites so that any participants are able to access the results via their treating clinician.

### Oversight groups and committees

The trial is overseen by the TSC. No DMEC will be convened; this role will be assumed by the TSC.

## Additional file

Additional file 1: Model Consent Form. (PDF 111 kb)

## Abbreviations

BCN: Breast Care Nurse
CBT: Cognitive Behavioural Therapy
CEAC: Cost-Effectiveness Acceptability Curves
CRF: Case Report Form
CSRI: Client Service Receipt Inventory
DCIS: Ductal Carcinoma in Situ
DMEC: Data Monitoring and Ethics Committee
EQ-5D-5 L: European Quality of Life-5 Dimensions-5 Levels
FACT-B: Functional Assessment of Cancer Therapy for Patients with Breast Cancer
FACT-ES: Functional Assessment of Cancer Therapy for Patients with Endocrine Symptoms
GAD-7: Generalised Anxiety Disorder Questionnaire
GCP: Good Clinical Practice
HFBBS: Hot Flush Beliefs and Behaviour Scale
HFNS: Hot Flush and Night Sweats
HFRDIS: Hot Flash Related Daily Interference Scale
HRT: Hormone Replacement Therapy
ICER: Incremental Cost-Effectiveness Ratios
ISF: Investigator Site File
NCRI: National Cancer Research Institute
PHQ9: Patient Health Questionnaire-9
PI: Principal Investigator
PIS: Participant Information Sheet
PSQI: Pittsburgh Sleep Quality Index
QALY: Quality Adjusted Life Years
QoL: Quality of Life
RCT: Randomised Controlled Trial
REC: Research Ethics Committee
RN: Research Nurse
SAE: Serious Adverse Event
SCTU: Southampton Clinical Trials Unit
SSRI: Selective Serotonin Re-uptake Inhibitors
SUSAR: Suspected Unexpected Serious Adverse Reaction
TM: Trial Manager
TMF: Trial Master File
TMG: Trial Management Group
TSC: Trial Steering Committee
