# Supplementary material for: MENOS4 trial: a multicentre randomised controlled trial (RCT) of a breast care nurse delivered cognitive behavioural therapy (CBT) intervention to reduce the impact of hot flushes in women with breast cancer: Study Protocol
Source: BMC Womens Health. 2018 May 8;18:63. doi: 10.1186/s12905-018-0550-z (PMC5941327; doi:10.1186/s12905-018-0550-z)
Supplement: Supplementary file 1 — Model Consent Form. (PDF 111 kb) [file 12905_2018_550_MOESM1_ESM.pdf]

(Form to be on headed paper)

MENOS 4: A multicentre randomised controlled trial of a breast care nurse delivered cognitive behavioural therapy (CBT) intervention to reduce the impact of hot flushes in women with breast cancer

Participant Identification Number ☐☐ - ☐☐

Please **write your initials** in each box to confirm that you have read and agree to the following statements:

1	I confirm that I have read the information sheet dated 19th April 2017 (version 3) for the above study. I have had the opportunity to consider the information, ask questions over the phone or face to face and have had these answered satisfactorily.	Initials
2	I understand that my participation is voluntary and that I am free to withdraw at any time without giving any reason, without my medical care or legal rights being affected.	Initials
3	I consent to be contacted by the research team as is required for the study, and I understand that my name, postal and email addresses and phone number will be stored securely and will not be revealed to anyone outside of the study team.	Initials
4	I consent to be contacted during the study by the research team to be invited to discuss my experiences of being part of the MENOS 4 Study (optional).	Initials
5	I understand that group CBT sessions will be recorded and listened to by a Clinical Psychologist from the trial team. I consent to be recorded if I am randomised to receive CBT.	Initials
6	I understand that relevant sections of my medical notes and data collected during the study may be looked at by individuals from regulatory authorities or from the NHS Trust, where it is relevant to my taking part in this research. I give permission for these individuals to have access to my records.	Initials
7	I understand that the information collected about me may be used to support other research in the future, and may be shared anonymously with other researchers.	Initials

8 I agree to my General Practitioner being informed of my participation in the study. In the unlikely event that I am hospitalised during the trial for unforeseen reasons, I give my consent for the trial team to contact my GP to ascertain the cause and duration of my admission. Initials

9 I agree to take part in the above study. Initials

Name of Participant	Date	Signature
Name of Person taking consent	Date	Signature

When completed (2 copies):
1 for researcher site file
1 (original) to be kept in medical notes

Please return this with your completed questionnaire and diary

(Form to be on headed paper)

MENOS 4: A multicentre randomised controlled trial of a breast care nurse delivered cognitive behavioural therapy (CBT) intervention to reduce the impact of hot flushes in women with breast cancer

Participant Identification Number [ ][ ] - [ ][ ]

Please **write your initials** in each box to confirm that you have read and agree to the following statements:

1	I confirm that I have read the information sheet dated 19th April 2017 (version 3) for the above study. I have had the opportunity to consider the information, ask questions over the phone or face to face and have had these answered satisfactorily.	Initials
2	I understand that my participation is voluntary and that I am free to withdraw at any time without giving any reason, without my medical care or legal rights being affected.	Initials
3	I consent to be contacted by the research team as is required for the study, and I understand that my name, postal and email addresses and phone number will be stored securely and will not be revealed to anyone outside of the study team.	Initials
4	I consent to be contacted during the study by the research team to be invited to discuss my experiences of being part of the MENOS 4 Study (optional).	Initials
5	I understand that group CBT sessions will be recorded and listened to by a Clinical Psychologist from the trial team. I consent to be recorded if I am randomised to receive CBT.	Initials
6	I understand that relevant sections of my medical notes and data collected during the study may be looked at by individuals from regulatory authorities or from the NHS Trust, where it is relevant to my taking part in this research. I give permission for these individuals to have access to my records.	Initials
7	I understand that the information collected about me may be used to support other research in the future, and may be shared anonymously with other researchers.	Initials

8 I agree to my General Practitioner being informed of my participation in the study. In the unlikely event that I am hospitalised during the trial for unforeseen reasons, I give my consent for the trial team to contact my GP to ascertain the cause and duration of my admission. Initials

9 I agree to take part in the above study. Initials

Name of Participant	Date	Signature
Name of Person taking consent	Date	Signature

Please keep this copy for your records
